# IUFoST/CIFST hold an Extraordinary Scientific Roundtable on COVID-19 and Food Safety

**DOI:** 10.1038/s41538-020-0068-2

**Published:** 2020-04-30

**Authors:** Gerald G. Moy

**Affiliations:** Food Safety Consultants International, 6 Chemin des Peutets, 1253 Vandoeuvres, Switzerland

**Keywords:** Agriculture, Economics

On 21 March 2020, an Extraordinary Scientific Roundtable was held concerning the COVID-19 and its implications for food safety as well as challenges and possible solutions for the food sector. In collaboration with the Chinese Institute of Food Science and Technology (CIFST), the Roundtable was convened under the auspices of the International Union of Food Science and Technology (IUFoST), which is the global scientific organization representing food scientists, technologists and engineers. The purpose of the Roundtable was to raise the awareness of the relationship between the coronavirus and food safety and, with the help of experts, to reach a consensus on how the global food community might cope with the challenges of a pandemic. The Roundtable included scientists from international and national health emergency response agencies as well as a representative of the food industry.

The Chair of the Roundtable, Dr Fereidoon Shahidi who is also the Chair of the IUFoST Scientific Council and Professor in the Department of Biochemistry, Memorial University in Newfoundland, Canada, welcomed the participants by noting that many countries have already ordered the complete closure of businesses, except for food stores and other essential services. Obviously, food stores are only the last link in the complex food supply network, with producers, importers, processors, manufacturers and distributors all being essential to provide food to home-bound populations. He stated that IUFoST was working to provide relevant guidance to the food industry during this global health crisis. To this end, the IUFoST Board established a new Task Force that will concentrate on “best practices” in the food sector during the pandemic that can be shared among all colleagues and interested parties worldwide. As the global voice of food science and technology, it is imperative that IUFoST uses its contacts around the world to quickly identify innovative and workable solutions to help others in this critical time. Anyone wishing to offer suggestions and insights in this regard should contact him at fshahidi@gmail.com with a copy to secretariat@iufost.org.

The Co-Chairs of the Roundtable were Dr Junshi Chen, Chief Adviser, China National Center for Food Safety Risk Assessment and member of the Chinese Academy of Engineering, and Dr Pingfan Rao, IUFoST Past President and Professor and Director of CAS.SIBS-Zhejiang Gongshang University Joint Center for Food and Nutrition Research in Hangzhou. They served as moderators for the discussion panel.

The first speaker was Dr Peter Ben Embarek, Unit Head, Monitoring of Nutrition and Food Safety Events, International Food Safety Authorities Network (INFOSAN) at the World Health Organization (WHO) in Geneva, who stated that COVID-19 appears to be a zoonotic virus originating in bats, but is likely to have gone through an intermediate species to infect humans. He congratulated China on the fact that for the last few days, not a single new domestic case was reported. He stated, “This is an amazing achievement, and also a message of hope for the rest of the world, showing that this outbreak can be brought under control, even though it might at times seem overwhelming. Systems can fight it back and put the virus under control. It is not a mission impossible; it is feasible, and several countries have shown that it can be brought back under control with the right measures.”

Dr Junshi Chen noted that during the extensive outbreak in China, there has never been any report of the transmission of the virus through food. Based on current epidemiological data, therefore, the coronavirus is not considered to be foodborne. He also noted that the United States Centers for Disease Control and European Centre for Disease Control have stated that there was no evidence that food items imported from China, in accordance with related regulations, posed a risk of spreading COVID-19 in their countries. He stated, “In normal cases, all cooked food will not contain any viable virus. But for cautionary purposes, it is recommended not to consume raw or undercooked animal products.” WHO has confirmed that the disease is spread from person to person through small droplets from the nose or mouth when a person with COVID-19 coughs or exhales. This conclusion has been endorsed by many national health authorities. The potential presence of the virus on food packaging was not currently considered sufficient to cause infection. The discussants agreed that the effective methodology used in Wuhan to control the disease should be emulated as much as possible, i.e., identifying, admitting and isolating confirmed COVID-19 cases and following up with all known contacts. However, the capacity to isolate infected persons and to treat severe cases is critical.

Dr Ding Gangqiang, Vice President of CIFST and Director of the Nutrition and Health Department within the China Centers of Disease Control, was deployed in Wuhan and noted that hotels were used for people who were at risk of infecting others. He also observed that the nutritional impact from isolating consumers indicated that, while energy-level intakes remained constant, intakes. The subject verb needs to agree of vitamins, minerals, plant-based protein and essential fatty acids were insufficient. In addition, he noted that great reduction in physical activity was observed, which may have longer-term effects on obesity and non-communicable diseases. Furthermore, as a group, the elderly did not pay sufficient attention or take adequate measures to change or control diet-related risk factors. He emphasized that the provision of safe and nutritionally balanced food to frontline workers, i.e. doctors, nurses and other healthcare providers, was considered essential and that cooked food was provided to them throughout the crisis in spite of numerous difficulties.

Dr Patrick Wall, member of the Irish COVID-19 National Emergency Response and Professor of Public Health at the University College Dublin, noted that primary food production may not be severely affected because of its geographic dispersal away from population densities, but harvesting and transport may be problematic. In this regard, the timely supply of both animal feed and ingredients in the food supply chain may be threatened, particularly if imported. The present reliance on specific markets, products and distribution channels was not likely to be robust enough to respond to anticipated disruptions. In addition to tourism and hospitality sectors, commercial catering, including restaurants and other food service establishments, will be severely disrupted with serious human and economic costs. Dr Wall noted that food businesses must keep their staff healthy if they are to function and this includes maximizing physical distancing at work. Physical distancing in the workplace must be accommodated, especially on the factory floor. In some production lines where physical distancing is not possible, the spread of the virus can decimate the workforce. Some processing companies are not only segregating workers by zoning but also temporally. If a zone or shift goes down, only a small number of people have to be excluded from working. Employees who contracted the virus need to be enabled and empowered to report their illness and remain at home. As a result, labor shortages may occur and solutions should be put in place, possibly through e-technology, regarding recruitment and training of new staff. Workers from commercial catering as well as from tourism and hospitality industries with food experience, can be redeployed into other parts of the food industry. A priority must still be placed on maintaining current levels of food safety, including preparedness for food recalls.

The participants agreed that the food industry needs to urgently respond to various disruptions to ensure that an adequate, safe and nutritious food supply is maintained for the local community. This was especially critical for large urban populations in developing countries. Food businesses need be given the tools and training to be able to support diverse food supply chains. The food industry needs to respond to rapid changes in the products that consumers need and want, which may require modifications in raw materials and/or processing to ensure product availability. Staffing in the food industry is essential if the food supply is to be maintained.

Dr Luo Yunbo, Honorary Vice President of CIFST and Director of the Research Centre for Special Food at the China Agricultural University in Beijing recognized that this crisis shows again the real importance of food safety—good hygiene practices must be practiced by everyone, not just those in the food industry, but also by the consumer. Food handlers, from industry to consumer, should be reminded to follow the WHO Five Keys to Safer Food and particularly, to wash their hands. The disruption of the food supply chain poses new and perhaps unexpected food safety risks, particularly for perishable foods. Information about food safety needs to be communicated in an easily understandable, scientific and thoughtful manner to all stakeholders in the food chain, from producers to consumers, particularly in these uncertain and chaotic times. He noted that the development of safe and effective foods to promote immune function should be a priority for the food industry and governments. This may include foods for medical use by the elderly population as well as other vulnerable groups.

E-commerce in food was undergoing tremendous growth as consumers responded to lockdowns, physical distancing and fear of crowds. Ms Liz Duffy, Vice President for Omnichannel Compliance for Walmart Global eCommerce stated, “In the e-commerce space, in particular, consumers solely shop online or primarily shop online, or spend a significant amount of time online. They are getting inundated with information around the outbreak and unfortunately, a lot of that information is not founded in science. They are looking at social media sites and other information sources to identify information around the outbreak, and so I think it is important that, as we can interact with consumers, to make sure that they are getting the most accurate information.” The participants agreed that it was essential to educate consumers and provide them with the accurate and timely information that they need to make good decisions. Consumer behavior will also distort the food supply chain by hoarding and panic buying that are often fueled by false information. The food industry needs to allay consumers’ anxiety through clear, scientific, user-friendly messages that do not contain false claims.

Dr Pingfan Rao noted the post-pandemic phase may result in major reviews of food systems with special emphasis on resilience. This also affords the opportunity for changes in agri-food systems that may make better use of locally produced foods. A paradigm shift regarding safe food practices should be reinforced by promoting good food safety habits that were developed during the pandemic. The food science and technology community should contribute to the recovery of the food sector along with other sectors. Several participants commented that food scientists and technologists should have a stronger role in government policy and contingency planning to ensure the resilience of the food supply chain in responding to future pandemics, including civil emergencies.

In summarizing, Dr Shahidi thanked the participants for their informative comments and suggestions and he hoped that this would be useful to the food science and technology community as well as the food industry and governments. He noted that the COVID-19 pandemic has posed an unprecedented challenge to the food sector, especially as many people may need to be quarantined for long periods of time. However, he had no doubt the food industry, aided by the food science and technology community, would meet this challenge and provide sufficient, safe and nutritious food to the world’s population. He again reiterated the commitment of IUFoST and its national Adhering Bodies to take all measures necessary to contribute to this goal.

IUFoST plans further roundtables to discuss matters of concern related to the coronavirus.

